# Opportunities for gender transformative approaches in a community-based drowning reduction program in Bangladesh

**DOI:** 10.1186/s12939-020-01226-z

**Published:** 2020-07-01

**Authors:** M. Gupta, A. Rahman, N. C. Dutta, D. Nambiar, R. Ivers, J. Jagnoor

**Affiliations:** 1 The George Institute for Global Health Australia, University of New South Wales, Sydney, Australia; 2 Centre for Injury Prevention and Research, House B 162, Road 23, New DOHS, Mohakhali, Dhaka, 1206 Bangladesh; 3 The George Institute for Global Health India, 311-312, Third Floor, Elegance Tower, Plot No. 8, Jasola District Centre, New Delhi, 110025 India; 4 School of Public Health and Medicine, Faculty of Medicine, UNSW Australia, Samuels Building, Botany Street, Kensington, 2052 Australia

**Keywords:** Gender analysis, Implementation science, Drowning, Child health, Community health workers, Program sustainability

## Abstract

**Background:**

Community-based programs in rural low-and middle-income country settings are well-placed to conduct gender transformative activities that aid program sustainability and catalyse wider social change, such as reducing gender inequities that in turn improve health outcomes. The Anchal program is a drowning prevention intervention for children aged 1–5 years old in rural Bangladesh. It provides community crèche-based supervision delivered by local trained paid-female volunteers. We aimed to identify the influence of the Anchal program on gender norms and behaviours in the community context, and the effects these had on program delivery and men and women’s outcomes.

**Methods:**

Qualitative in-depth interviews, focus group discussions and observations were conducted with program beneficiaries and providers. Gender outcomes were analysed using FHI 360’s Gender Integration Framework.

**Results:**

The Anchal program was found to be a gender accommodating program as it catered for communities’ gender-based roles and constraints but did not actively seek to change underlying beliefs, perceptions and norms that led to these. The program in some cases enhanced the independence and status of female community staff. This changed perceptions of communities towards acceptable levels of physical mobility and community involvement for women. Conversely, gender affected program delivery by reducing the ability of female supervisory staff to engage with male community leaders. The double burden of wage and household labour carried by local female staff also limited performance and progression. Gender-based constraints on staff performance, attrition and community engagement affected efficiency of program delivery and sustainability.

**Conclusions:**

The Anchal program both adapted to and shaped community gender norms and roles. The program has well-established relationships in the community and can be leveraged to implement gender transformative activities to improve gender-based equity. Health programs can broaden their impacts and target social determinants of health like gender equity to increase program sustainability and promote equitable health outcomes.

## Highlights

- Community-based programs in lower-middle income country contexts have a bi-lateral relationship with contextual gender norms, roles and relationships
- The Anchal drowning reduction program in Bangladesh has affected gender-based norms relating to women’s employment and physical mobility
- The Anchal program is in turn affected by cultural norms and must cater to these, such as hiring only women in child caregiving roles and recruiting fewer female supervisory staff due to mobility and cultural constraints
- Community-based programs such as Anchal are well-embedded in communities and have the opportunity to be gender transformative and actively seek to change harmful or limiting gender norms and roles

## Background

To enhance uptake and sustainability of community based health programs it is important to highlight the suite of additional beneficial outcomes, such as impacts to wider social norms and other health indicators. These broader outcomes may affect acceptance and implementation of a program, and hence its sustainability [1–3]. Community-based programs build close relationships with people in their context, and can comprise negotiation and sometimes influencing of gender norms, perceptions and behaviours [4, 5]. How women and men differently perceive, interact with and respond to programs affects delivery and effectiveness [5, 6]. Including gender analysis in evaluations can therefore reveal how involvement or exposure to a program may be affecting gender in implementation contexts [7–9].

While community-based programs no doubt affect cultural gender norms, gender-based roles may in turn affect program delivery and sustainability, especially in cases where local women are agents of change or program beneficiaries. Understanding the gender-related constraints local staff face may improve retention and wider community acceptability [10, 11]. Further, programs may also change community norms towards women’s employment over time, improving access to a capable workforce [12]. Many rural Low-And Middle-Income Country (LMIC) child programs also engage extensively with mothers, the main caretakers of children. Identifying how mothers are affected by gender-based attitudes, such as towards mobility, decision making at home and engagement with men, may reveal insights for programs on improving accessibility [13, 14].

The Anchal program is a community-based drowning reduction intervention in rural southern Bangladesh. It hires local women to supervise groups of children in a secured space during daytime when they are most at risk of drowning. Rural communities in Bangladesh typically follow gender-based norms and roles for men and women [15]. Women are primarily responsible for household work and child-rearing, while men engage in income-earning work requiring frequent travel; women experience cultural restrictions on mobility, access to formal employment and participation in leadership roles [16–18]. Bangladesh ranks 142 out of 167 countries on the Women Peace and Security Index, with particularly low scores on indicators relating to equitable access to education, employment and finances [19].

We conducted a gender analysis of the Anchal program aimed at identifying and assessing the bi-lateral relationship between the program and effects on gender norms, roles and relationships. We identified program influences on gender-related context, and how gender influenced program delivery and sustainability. The detailed protocol for this study has been previously published [20].

## Methods

Qualitative methods were used to develop an in-depth understanding of relationships between the program and contextual gender norms, roles and behaviours in communities. A range of key stakeholders were engaged to ensure perspectives from both program providers and beneficiaries.

### Program characteristics

A drowning mortality survey in Bangladesh found high rates of drowning at 121 per 100,000 children 1–4 years old [21]. In the southern Barishal Division, these rates are higher at 262.2 per 100,000 children due to greater presence of water near households, poor water protection infrastructure, and limited health systems reach. The region is rural, and the population is largely of lower socioeconomic condition, with many households having four or more children. Most adults work in fishing or agriculture. Most of the population are Muslim and speak the national language Bangla, though small minorities of Rakhine people live in the area [22].

The Anchal program was introduced in 2016 for the purposes of drowning prevention by a large non-governmental organisation, the Centre for Injury Prevention and Research, Bangladesh (CIPRB), based on a World Health Organization recommendation to keep children in crèches during at-risk hours [23]. CIPRB runs 400 Anchal centres across its intervention areas in the Barishal Division. The program provides safe and supervised space to children during the time of day when they are most at risk of drowning and early childhood development activities are part of the program. Each Anchal centre is managed by a locally-trained Anchal *Maa* (caregiver) and Anchal Assistant, with 20–25 children aged 1–5 years old under their care from 9 am to 1 pm, 6 days a week. The centres are most often located in the home of the Anchal Maa.

Anchal staff (Anchal Maas and Anchal Assistants) are directly managed by Supervisors employed by the program provider. Of 14 supervisors, three are women. Each Supervisor oversees between 25 and 30 centres. Anchal Maas are also provided ongoing training and support from female Anchal Mentoring Officers (AMOs) in monthly cluster meetings. See Additional file 1 for a diagram of the program’s organisational structure.

Within each community, program implementers established a Village Injury Prevention Committee (VIPC) comprised of leaders and influential locals. The VIPCs oversee program operations, contribute to community outreach and facilitate Anchal Maa and Assistant recruitment. At the lowest government ward level, Union Injury Prevention Committees (UIPCs) engage government and community leaders bi-annually to update on program progress and gather support.

See Additional file 2 for a logic model of the Anchal program, describing its key components.

### Framework

The Anchal program is implemented in a rural community setting and is impacted by its social context, including existing gender norms, roles and relationships. We sought to better understand this context using the Gender Integration Framework [7], which allows for the analysis of the roles men and women play within the program and wider community. The framework has been applied to community-based interventions in LMIC contexts, including programs that seek to address gender-based violence, HIV and food insecurity [24, 25].

The framework was used to analyse how gender interacted with program delivery and affected opportunities, needs, constraints and relationships across the five key domains outlined in Table 1. The domains capture a breadth of possible bi-directional effects between the Anchal program and gender norms, roles and relationships.

**Table 1:** Gender Integration Framework domains [7]

Domain	Examples of key gender relations, barriers and opportunities
1. Access to resources	Education, information, services, employment, benefits, freedom of movement, transport
2. Knowledge, beliefs, perceptions	Beliefs about capabilities, self-efficacy and confidence, acceptable behaviour and value in society, child safety and protection
3. Practices and participation	Autonomy and time to participate both within the home and in the community, types of activities and practices
4. Legal rights and status	Employment contracts and rights, biases in governance and policy at program and institutional level
5. Power	Autonomy, household financial control, control over resources, decision making within the household and in the community

### Data collection

Data collection was of a qualitative nature with key informant In-Depth Interviews (IDIs), Focus Group Discussions (FGDs) and observations. While IDIs provided insights into individual perspectives, FGDs provided information on community dynamics and norms, and involved between 6 and 8 participants. Observations allowed for a deeper understanding of program settings and interactions between providers, beneficiaries and the community. These data sources were triangulated to provide a comprehensive picture of gender effects. A total of 57 IDIs, four FGDs and three observations were conducted engaging 41 men and 48 women. See Table 2 for a summary of all participants.

**Table 2:** Summary of participants

Participant Role	IDIs		FGDs	
	Male	Female	Male	Female
*Program beneficiaries*				
Parents of enrolled or graduated children	1	3	2	–
Parents of drop out children	3	3	–	–
Parents of unenrolled children	3	3	–	–
*Program providers*				
Anchal Maa	–	6	–	1
Anchal Assistant	–	6	–	1
Early Childhood Development Specialist	–	1	–	–
Anchal Mentoring Officers	–	5	–	–
Supervisors	3	2	–	–
Area Coordinators	3	–	–	–
Project Coordinator	1	–	–	–
Village Injury Prevention Committee (VIPC) members	7	2	–	–
Program headquarters staff	4	1	–	–
**Total**	**25**	**32**	**2**	**2**

Qualitative methodology was guided by the Consolidated Criteria for Reporting Qualitative Research (COREQ) (see Additional file 3), which details steps for reporting qualitative research using IDIs and FGDs [26]. Data was collected by trained data collectors in Bangla across all three intervention areas. Qualitative data collection of all types (IDIs, FGDs and Observations) was divided into two phases. Approximately half the data was collected in Phase 1 from September to October in 2018 and was analysed and used to inform key themes for investigation in Phase 2 from March to April 2019. Findings that were found to be unclear or unusually homogenous in Phase 1 were probed further in Phase 2.

Anchal Supervisory staff introduced data collectors to the communities, who engaged local leaders to gain appropriate permissions before approaching participants. All participants were recruited and interviewed face to face. All IDIs and FGDs were audio recorded and held in private locations that were accessible for the participant and lasted between 30 min and 1.5 h. Only data collectors and participants were present. Less than 10% of participants refused to participate (*n* = 3) once initially approached. Data collection for each participant role ceased once thematic saturation was reached in Phase 2.

### Sampling and selection criteria

Perspectives were gathered from both program beneficiaries and program providers. To understand program beneficiary perceptions, convenience sampling was used to recruit three types of parents in the community: those who had enrolled in the program, those who had enrolled and dropped out of the program, and those who had been approached by Anchal program staff but refused to enroll. This provided information on gender-related barriers and enablers to participation. They also provided insights into wider community responses to the program. Both male and female parents were equally engaged to identify differences in perspectives by genders.

Program providers included all those who implemented the Anchal program and oversaw its activities. In both phases, purposive sampling was used to recruit program staff who provided specific insights to gender-related program delivery challenges and community responses. These included community-level staff such as Anchal Maas and Assistants, community-level VIPC members who supported on-ground implementation, and staff employed by CIPRB to monitor the program and manage background processes including Anchal Mentoring Officers, Supervisors and program headquarter staff. Community-level staff and VIPC members had close relationships with the wider community and were asked to provide insights on responses to gender-based roles and norms being catered to or challenged by the program. Program providers were privy to responses from local government and community leader responses, and how gender norms and roles affect program delivery. All Anchal Maas, Anchal Assistants and AMOs were female. Most other staff were male, bar two Supervisors, one headquarters staff and two VIPC members. IDIs provided individual experiences and perceptions of gendered effects andFGDs were held with community-level staff to capture community perspectives.

Additionally, three Anchal centre sessions and one Anchal Maa monthly cluster meeting were observed to understand engagement and interactions of providers and beneficiaries with the program and each other. No children participated in IDIs or FGDs due to their young age, but their actions and activities were observed as part of these observations. Data collectors noted their engagement with the centre activities and staff and levels of comfort.

#### Tools

The IDIs, FGDs and observations took a semi-structured format using interview/observation tools developed in accordance with the framework. The tools provided data collectors with questions and themes to explore, but provided them the opportunity to probe and record further detail when new ideas were expressed by participants. All tools were field tested before finalisation.

#### Data collectors

Three female and three male data collectors were recruited by the program provider for this project, led by and including ND. All were fluent in both English and Bangla and had previous Masters-level training in anthropology and social work with experience in qualitative data collection. They were trained by the research team on the study methodology and use of tools.

#### Consent

The aims of the study were described to all participants. Participants were informed that data collectors were hired by the implementing organisation as independent interviewers. Informed written consent was sought from all participants. Written consent for child participants was sought from their parents or guardian. Where literacy was a barrier, data collectors verbally described the study as per the Participant Information Statement and obtained fingerprints for consent.

#### Ethics approval and consent to participate

Local ethical approval from the Centre for Injury Prevention and Research, Bangladesh Ethical Review Committee was granted (Memo no: CIPRB/ERC/2017/24). Ethical approval was also obtained from the University of New South Wales Human Research Ethics Committee (HC: 180608).

All participants were informed on study objectives and freely provided verbal or written consent to participate and be audio recorded (as depending on participant’s literacy). Participant Information Statements were provided in Bangla where required.

### Analysis

All qualitative data were transcribed in Bangla and translated into English for analysis by professional transcription and translation agencies. A fluent Bangla and English speaker completed a quality check on all documents to ensure translation and transcription was accurate. Transcripts or findings were not returned to participants due to logistical difficulties and low literacy rates in the region.

Analysis of the transcribed qualitative data was assisted by NVivo 12 software [27]. Deductive thematic analysis was conducted using the Gender Integration Framework [28, 29]. A priori codes were based on the framework domains and sub-codes under these were generated based on findings. Transcripts were also coded for qualitative method type (IDI, FGD and Observation) and points of convergence and divergence were identified to triangulate findings and identify differences between community and individual-level perceptions and effects [30]. Two independent teams of researchers headed by MG and NCD initially coded the data before discussing discrepancies and identifying final themes. See Additional file 4 for Phase 1 and 2 codebooks.

This analysis allocated the program along the Gender Integration Continuum [7], which described whether the program was gender exploitative, accommodative or transformative. Exploitative programs are those that take advantage of and perpetuate harmful gender norms to meet outcomes, such as reinforcing harmful gender roles or practices. Accommodative programs may acknowledge or cater to gender norms, but do not seek to change them. Gender transformative programs actively seek to change harmful gender norms to improve outcomes for both men and women by targeting the underlying causes for gender disparities, such as by shifting attitudes that prevent men from being caregivers and women from being bread earners. Often for men and women to have equitable outcomes, differential support and strategies are required.

## Results

Bi-directional effects of the Anchal program and gender were found. The program was influencing gender norms, roles and relationships in the communities, while also adapting its delivery to cater to these. The results are discussed below as per the types of gender effects along the framework domains. No findings were reported against the domain of *Legal rights and status* as this refers to impacts of the wider rights and status of women under law.

### Access to resources

Involvement in the program led to changes in access to resources for Anchal Maas and Anchal Assistants hired in the community. As part of the program, Anchal Maas and Assistants opened their own bank accounts to receive their salary.

Women in communities previously had few opportunities for formal employment due to constrained economic conditions and cultural limitations on appropriate work for women. Employment in the Anchal program provided a rare opportunity for women to earn an income.

*“The Anchal Maas or Assistants … find it a good opportunity. Because community people, especially females, work at home. They do not have access to any income generating activities through which they can earn.” - [Supervisory Staff, Male]*

With access to income Anchal Maas and Assistants contributed to family expenses and businesses which reduced financial stress. Anchal Maas purchased fertiliser for their farms, materials for trade or invested in goats and chickens. They contributed to better education for their children with admissions into private schools and tuitions. Greater access to resources such as mobile phones, refrigerators, TVs and other electrical devices improved their overall quality of life.

*“She makes assets with her earnings, such as making gold ornaments…They also utilize the money in their family businesses.” - [Anchal Mentoring Officer, Female]*

*“I have children and I spend my money on them. I admitted my son in a better private school and their fee is costly.” - [Anchal Maa, Female]*

In addition to material changes for Anchal Maas and Assistants, access to income improved financial independence. Anchal Maas became less reliant on male family members for money and showed more discretion and efficiency when spending. For example, Anchal Maas were able to quickly purchase emergency medicine if their child was sick.

*“Previously I would have to demand from my husband if I wanted to purchase anything and ask him several times. Now I can buy anything that I like to buy. There is no problem now.” - [Anchal Maa, Female]*

### Knowledge, beliefs and attitudes

Within communities, women were considered responsible for household domestic duties and child rearing as per prevailing gender norms. The Anchal program was acceptable to communities as it catered to these gender norms by largely taking place in the women’s homes and involving the care of children.

*“Still, there are some who don't allow their daughters and daughters-in-law to work outside the home, but as they are working inside the home, they [the Anchal Maa’s family] accept it.” - [Supervisory Staff, Female]*

Although the Anchal program upheld norms pertaining to women’s roles, it simultaneously influenced the breakdown of constructive attitudes towards mobility. Culturally, most women in these communities did not travel beyond a few homes alone due to gender-based norms and fears for safety. Anchal Maas, however, travelled within and between communities for training, child enrolment and community meetings. Many Anchal Maas and Assistants’ families were initially concerned about this outside exposure but became accepting as Anchal staff became settled into their roles and fears around safety dispersed with experience. Anchal Maas gained more independence and access to resources as they were free to travel outside the community to places such as markets without taking permission. Anchal Maas and Assistants appeared to be self-assured in their right to independence and chose to ignore negative perceptions from their communities around their mobility.

*“She goes house to house now. She would not go outside of the home earlier because of criticism, as she is a wife. Now she does not think about that criticism.” - [Supervisory Staff, Female]*

Involvement in the program also challenged the norms preventing women from entering formal employment. Families became more accepting of women working and interacting with program implementing staff, including men. Some Anchal Maas were able to access additional employment because of the knowledge, skills and experience they gained through the Anchal program. Anchal Maas and Assistants developed their own identity as teachers and advisors in addition to their role as a mother, wife or daughter-in-law.

*“Nobody gave me any attention before. But now I have become a President for one NGO a few days ago. I go for a meeting every Friday. We receive 20,000 taka and I have the responsibility of managing this money.” - [Anchal Maa, Female]*

These changing perceptions around women’s work influenced the perceived value of educating girls. Anchal Maas also reported that community girls they interacted with felt encouraged to continue their education so they may also 1 day find employment. Lack of exposure to working women also meant that communities may have viewed women as unable to effectively engage with formal work. However, according to both program providers and community members, exposure to the Anchal program shifted community beliefs around the capability of housewives in conducting complex tasks and contributing in the wider community.

*“The people of the village think that housewives can’t do anything. But now, they realize that if we let her train a little then she would be able to do a big job.” - [Anchal Maa, Female]*

Gender attitudes affected program monitoring staff. Female Supervisors experienced initial difficulty in engaging with male community leaders due to limiting norms around interaction between genders and little community exposure to women in leadership positions. Compared to male Supervisors, female Supervisors sometimes required more time to build rapport with community leaders and gain permission for implementing the Anchal program as leaders were slower to trust and engage productively with them. However, female Supervisors were better able to access households and engage mothers at a community level than male Supervisors due to cultural barriers on interactions between members of opposite genders.

*“The male Supervisors are better able to connect with the decision makers. Suppose a Supervisor met a chairman at a tea shop in the evening and they sat together and had some tea and a chat. My female Supervisors can’t do it…The females get more access to connect with the local people but the males connect better with the decision makers.” - [Supervisory Staff, Male]*

Hiring women local to the area as Anchal Mentoring Officers or Supervisors mitigated some of these challenges for female program monitoring staff, as they had pre-existing relationships with the communities.

*“… the AMOs of Kalapara are new in the job. I am working for a long time. I was an administrator in a local NGO...It is better for me.” - [AMO, Female]*

### Practices and participation

Employment in the Anchal program led to limited changes in the roles of Anchal Maas and Assistants at home. Anchal Maas and Assistants’ responsibilities were still constrained by their defined domestic gender roles and their lower position in the household hierarchy. Anchal Maas and Assistants were expected to carry a double burden, being responsible for both domestic work and child rearing. Anchal Maas and Assistants worked long hours to ensure all their work was completed. Some received support from other women in their family, but often only in return for sharing their income. A major reason for Anchal Maa and Assistant drop out was difficulty in managing responsibility at home with work in the Anchal program.

*“They have to make the baby’s food, take care of the father-in-law, her husband’s needs, demands of the office. She does a lot of jobs. And we have to create a suitable environment for her to do her jobs well and support her.” - [Supervisory Staff, Male]*

Gender roles also reduced the ability of the program to engage effectively with fathers of Anchal children. Although fathers were usually the final decision makers on children’s attendance in the Anchal program, they were more likely to be away for work and rarely available for engagement due to their roles as family breadwinners. Many fathers in the region are fishermen who are absent for weeks at a time. This often slowed initial enrolment. Fathers were also less likely to be educated on the Anchal program activities and injury prevention methods through parent meetings.

*“If you analyse our parent meetings, VIPC meetings, any kinds of meetings that we run, the fathers are not found. The number of fathers is very low. All the mothers remain present. Because most of the people in this area are fishermen.” - [Supervisory Staff, Male]*

Male and female program monitoring staff reported being treated equally within the implementing organisation. However, the lack of differential support catering to gender differences meant that staff were not always able to produce the same outcomes. Female staff struggled with transport and mobility. As it was not culturally appropriate to ride their own motorbikes, female Supervisors were spending more time waiting for shared transport which impacted their productivity. They were also less able to respond to community problems or engagement opportunities that required night-time return travel due to safety concerns.

*“If the office provided vehicular support to female staff while working in the field it would be very helpful for us. It's needed only for females, males don't need it.” - [Supervisory Staff, Female]*

Conversely, male Supervisors faced difficulty in engaging with Anchal Maas and mothers due to cultural constraints on contact between genders. Male Supervisors were not able to provide Anchal Maas feedback or engage with mothers as effectively as female Supervisors. This was a problem in more conservative communities which follows the *parda* system, where women covered their faces in public and were unable to speak directly to men. This system prevented community women from talking to male Supervisors face to face. This adversely affected quality control of Anchal activities and community engagement for male Supervisors.

*“The women of this area are very sheltered. If our office staff go to them, they [the women] do not want to come out. They do not want to talk. There is a village where the women there will not even come out of the fence. Our supervisor talks with them from outside of their house.” - [Supervisory Staff, Male]*

### Power

Although the program encouraged shifts in thinking around women’s participation outside the home, their power to make autonomous decisions remained limited due to their lower place in household hierarchy and men’s roles as main decision makers. Family support was vital for Anchal Maas and Assistants to work. Anchal Supervisors spent considerable time ensuring family support before Anchal Maa and Assistant recruitment. In some instances, family members were invited to visit field offices to meet the larger team to gain their trust. Ongoing permission from Anchal Maas and Assistants’ in-laws, parents or husbands was essential for them to continue in the program. The family decision-making hierarchy where women were often at the bottom usually remained intact.

*“Because when a girl is married her parents don’t have any control over her. Her husband becomes everything for her. She has to do what her husband tells her to do.” - [Anchal Maa]*

*“Some family members like fathers and mothers-in-law also don’t keep their word. They say they will provide a room for the Anchal centre and will give support to Anchal Maa, but they don’t. At that time, it becomes very difficult for the Anchal Maa to continue and then they drop out. If the family doesn’t support the wife, it becomes difficult for that Anchal Maa.” - [Supervisory Staff, Male]*

### Varied experiences of Anchal Maas as compared to Anchal assistants

Anchal Assistants had lower educational qualifications and fewer responsibilities during centre hours, but were present with the Anchal Maa four hours a day and took sole responsibility of the children when Anchal Maas attended monthly cluster meetings. As their salaries were one third of Anchal Maas’, here was a less pronounced effect on access to material resources and financial independence. Many Anchal Assistants were motivated by the social cause of the work and some were foregoing other activities that may generate more income by working in the program.

*“You would be able to earn a lot working in tailoring. I would get at least 5000 taka wage. If I spend 3 to 4 hours here, I do not get that money…Society considers us [Anchal Maa and Assistant] good as we do great work...I like it very much.” - [Anchal Assistant, Female]*

For some Anchal Assistants, the low pay reduced their value in their family or community as it wasn’t considered enough for the number of hours worked. Some Anchal Assistants felt shame and lied about their earnings to others. This partially explained the higher dropout rate of Anchal Assistants compared to Anchal Maas reported by Supervisors.

*“I do the job, I get 1000 taka – but I do not tell anyone how much I earn. I tell them that I get about 2000 or 2500 taka otherwise there exists no honour for me.” [Anchal Assistant, Female]*

Anchal Assistants were also provided fewer opportunities for skill development and support from the Supervisors and AMOs, who concentrated their efforts on Anchal Maas. This led to lower engagement in the program and poorer development of their capabilities, possibly reducing their ability to access other employment or opportunities compared to Anchal Maas.

*“There she [Anchal Maa] learns so many things, so many tips. Since we are Assistant, we have to learn from her. That’s it. In addition to this, if something more is required to learn, then there should be one training for us as well. Suppose, if there will be one training for us in each month, then shouldn’t we participate in it? Couldn’t we learn so many things?” [Anchal Assistant, Female]*

### The Anchal program as gender accommodating

Based on the criteria for program allocations along the Gender Integration Continuum and the above findings, we found the Anchal program to be a gender accommodating program [7]. As per the Continuum, gender accommodating programs are those that acknowledge and cater to gender norms and roles, but do not actively seek to change those with negative health consequences. Anchal provided some opportunities to change gender norms and roles for community-based program providers through its regular activities, namely through recruiting Anchal Maas and Assistants. Communities became more accepting of women’s employment and mobility and Anchal Maas showed greater financial independence. However, the program did not actively implement strategies to address gender equity issues. Supervisors staff largely continued to function within gender-based constraints and roles, such as limiting norms around engaging with the opposite gender. Anchal Maas and Assistants also continued to have limited autonomy over domestic decision making. Mothers in the community were not exposed to any activities that challenged harmful gender norms despite opportunities to do so, such as through the regularly held parent meetings.

## Discussion

Our findings suggest that while the Anchal program was delivered in line with gender norms in rural Bangladesh, it did not actively seek to change them. Gender transformation usually occurs once a program is well embedded in the community [4]. At the time of the study the Anchal program had been operational for 2 years and strong ties had been developed between the program provider, community leaders and community members. Many long-term programs in rural LMIC contexts internationally have built such relationships by operating closely with communities over a number of years, such as those targeting HIV, child and maternal health, and are now in a position to take more active steps to transform gendered constraints [7]. For example, the Go Girl! Initiative (GGI) in Botswana, Malawi, and Mozambique set up community action groups to identify problems relating to HIV prevention and treatment, and helped girls and boys explore issues around gender [31]. Moving forward, delivery of the program may lead to changes in gender roles and perceptions that harm both men and women’s health and social outcomes. These changes may enable better integration with the community and improve sustainability. More data is required to better understand the link between gender transformation and long-term sustainability [32], and the Anchal program has an opportunity to contribute to this evidence-base.

At the time of the study, the Anchal program could not be considered a gender transformative program as it did not systematically employ strategies to change harmful or limiting gender norms and relationships that can improve program sustainability and the social position of women involved in it [7]. One opportunity to do this in the Anchal program could be increasing the visibility of women demonstrating leadership behaviours by engaging more female Supervisory staff and actively involving them in traditional male-dominated spaces such as UIPC meetings. Such visibility has been found to influence perceptions in the wider community in countries such as Pakistan and Afghanistan, including of women’s roles and participation [9, 10, 33, 34]. While the Anchal program has begun to change these perceptions by hiring female Supervisors and Anchal Mentoring Officers, further steps are required to support female Supervisors staff in performing equally to men and vice versa. The provision of tailored support to men and women based on their gender-based constraints may ensure better performance and further demonstrate the capability of women in these roles, such as was found successful in a Nigerian maternal health service program [35]. Possible strategies from other programs in LMICs that seek to address similar gender-based barriers to work performance include the provision of culturally appropriate motorcycles for female staff [36] and using films and performance theatre to challenge community perceptions around male and female interactions [37]. These strategies may improve retention, satisfaction of workers and build capabilities in communities, leading to greater sustainability. Increasing women’s mobility and leadership in decision-making also improves health outcomes for women as they have increased access to health services and information, and representation leads to attention and action on their needs [38–40].

Retention of Anchal Maas and Assistants may be improved by assessing the impact of the double burden of work and home duties. Solutions to improve conditions for Anchal Maas and Assistants at home may be brainstormed with the community, such as through gender sensitisation workshops and discussions which have been implemented in health programs internationally [41, 42]. In particular, the program may consider raising the pay of Anchal Assistants to meet community expectations, although the current amount is commensurate with minimum wage in Bangladesh. The social incentive to remain in the role may not be sufficient for Anchal Assistants in front of practical barriers related to wages. In other health programs around the world such as in Indonesia, Ethiopia, Kenya and Malawi, fair remuneration was found to be a key factor that improved empowerment outcomes of female community health workers and increased retention [9]. Raising pay may also improve perceptions towards the equal value of women’s work in a country with only 36% female workplace participation [43]. Economic empowerment has also been shown to improve women’s reproductive health outcomes in LMIC settings [44, 45].

As programs are impacted by their social context, the approach the Anchal program took in adapting delivery to gender roles, such as hiring women for childcare roles and more male Supervisors for leadership and community leader engagement roles, initially supported the implementation of the drowning program at the ground level. The program appeared to have acceptability in the view of communities who were generally supportive of hiring community women as Anchal Maas and Assistants and were also ready to accept some changes in their access to resources and mobility in return for their good work. As most community health workers in rural health programs in LMICs are women, being cognisant of gender attitudes and roles ensures that these workers are supported and retained [9, 46, 47].

Lastly, public policy structures and incentives can play a role in ensuring programs take gender transformative actions. For examples, cash incentives to parents to send girls to school have been effective in increasing girl engagement in developing countries, and similar policies can be applied to improve girl participation in physical education classes like swimming [48]. Policy can also hold organisations to account on employment practices, such as by renewing registrations on the basis of female staff quotas [49]. Given relationships generated by CIPRB with government-level stakeholders through implementation activities such as UIPC meetings, advocacy activities in this regard may be impactful at a larger scale.

### Limitations

No findings were reported on the effect of wider legal structures and context on gender as per the Gender Integration Framework domain of *Legal rights and status*. A longer study period and additional methods such as interviews with local government-level key informants may have revealed program influences on gender norms, roles and relationships mediated by women’s legal rights and status.

## Conclusions

The Anchal program is integrated in southern Bangladeshi communities and has been able to adapt to and shape gender norms and roles. However, further opportunities exist for the program to take strategic steps towards ensuring gender equity in social and health outcomes as well as program sustainability. Identifying and implementing initiatives that empower program provider staff, community staff and the wider community to challenge harmful gender-based norms and beliefs would move the program from being gender accommodative to gender transformative. These may include hiring and supporting supervisory female staff to change norms around working women, engaging communities in discussions relating to division of household work and child rearing between genders, and engaging males in childcare work. This has implications for men and women’s equity, health and financial outcomes, as well as for program success.

## Supplementary information

**Additional file 1.** Organisational chart of the Anchal program.

**Additional file 2.** Logic model for the Anchal program.

**Additional file 3.** COREQ (COnsolidated criteria for REporting Qualitative research) Checklist.

**Additional file 4.** Codebook

## Data Availability

The datasets used and/or analysed during the current study can be requested to the investigative team and would be made available are available on reasonable request.

## References

[1] Kruk ME, Porignon D, Rockers PC, Van Lerberghe W (2010). The contribution of primary care to health and health systems in low- and middle-income countries: A critical review of major primary care initiatives. Soc Sci Med.

[2] Klassen TP, MacKay JM, Moher D, Walker A, Jones AL (2000). Community-based injury prevention interventions. Futur Child.

[3] Druetz T (2018). Integrated primary health care in low- and middle-income countries: a double challenge. BMC Medical Ethics.

[4] Morgan M (2014). Measuring gender transformative change. Penang, Malaysia: CGIAR Research Program on Aquatic Agricultural Systems.

[5] Moser C (1993). Gender planning and development: theory, practice and training.

[6] Tannenbaum C, Greaves L, Graham ID (2016). Why sex and gender matter in implementation research. BMC Med Res Methodol.

[7] FHI 360 (2012). Gender Intergration Framework: How to integrate gender in every aspect of our work.

[8] Dworkin SL, Treves-Kagan S, Lippman SA (2013). Gender-transformative interventions to reduce HIV risks and violence with heterosexually-active men: a review of the global evidence. AIDS Behav.

[9] Steege R, Taegtmeyer M, McCollum R, Hawkins K, Ormel H, Kok M (2018). How do gender relations affect the working lives of close to community health service providers? Empirical research, a review and conceptual framework. Soc Sci Med.

[10] Mumtaz Z, Salway S, Waseem M, Umer N (2003). Gender-based barriers to primary health care provision in Pakistan: the experience of female providers. Health Policy Plan.

[11] Lehmann U, Sanders D (2007). Community health workers: what do we know about them?.

[12] Gopalan SS, Mohanty S, Das A (2012). Assessing community health workers’ performance motivation: a mixed-methods approach on India's accredited social health activists (ASHA) programme. BMJ Open.

[13] Feldhaus I, Silverman M, LeFevre AE, Mpembeni R, Mosha I, Chitama D (2015). Equally able, but unequally accepted: gender differentials and experiences of community health volunteers promoting maternal, newborn, and child health in Morogoro region, Tanzania. Int J Equity Health.

[14] Desai S, Johnson K. Women’s decision making and child health: familial and social hierarchies. In: Kishore, S. editor. A focus on gender: Collected papers on gender using DHS data. Calverton: ORC Macro; 2005. p. 55–68.

[15] Mahmud S, Shah NM, Becker S (2012). Measurement of Women’s Empowerment in Rural Bangladesh. World Development.

[16] Balk D (1997). Defying gender norms in rural Bangladesh: a social demographic analysis. Popul Stud.

[17] Sayem AM, Nury ATMS (2013). An assessment of attitude towards equitable gender norms among Muslim women in Bangladesh. Womens Stud Int Forum.

[18] Bridges S, Lawson D, Begum S (2011). Labour market outcomes in Bangladesh: the role of poverty and gender norms. Eur J Dev Res.

[19] Georgetown Institute for Women Peace and Security and Peace Research Institute Oslo (2019). Women Peace and Security Index 2019/20: Tracking sustainable peace through inclusion, justice, and security for women.

[20] Gupta M, Rahman A, Ivers R, Zwi AB, Hossain S, Rahman F, et al. Complexity in implementing community drowning reduction programs in southern Bangladesh: a process evaluation protocol. Int J Environ Res Public Health. 2019;16(6). 10.3390/ijerph16060968. 10.3390/ijerph16060968 PMC6466245 30889852

[21] Rahman A, Alonge O, Bhuiyan A-A, Agrawal P, Salam SS, Talab A, et al. Epidemiology of Drowning in Bangladesh: An Update. Int J Environ Res Public Health. 2017;14(5). 10.3390/ijerph14050488. 10.3390/ijerph14050488 PMC5451939 28475138

[22] Rahman A, Jagnoor J, Baset K, Ryan D, Ahmed T, Rogers K (2019). Vulnerability to fatal drowning among the population in southern Bangladesh: findings from a cross-sectional household survey. BMJ Open.

[23] Meddings D, Hyder AA, Ozanne-Smith J, Rahman A (2014). Global report on drowning: preventing a leading killer.

[24] Cothran D (2019). The Added Value of Gender Integration: Promising Practices from the Advancing Partners & Communities Project: Advancing Partners and Communities.

[25] Geleta EB, Elabor-Idemudia P, Henry C (2017). Scaling-up: gender integration and women’s empowerment in southern Ethiopia. Cogent Food Agric.

[26] Tong A, Craig J, Sainsbury P (2007). Consolidated criteria for reporting qualitative research (COREQ): a 32-item checklist for interviews and focus groups. Int J Qual Health Care.

[27] QSR International Pty Ltd (2018). NVivo qualitative data analysis software.

[28] Saunders B, Sim J, Kingstone T, Baker S, Waterfield J, Bartlam B (2018). Saturation in qualitative research: exploring its conceptualization and operationalization. Qual Quant.

[29] Brannen J (1992). Mixing methods: qualitative and quantitative research.

[30] Carter N, Bryant-Lukosius D, DiCenso A, Blythe J, Neville AJ. The use of triangulation in qualitative research. Oncol Nurs Forum. 2014;41(5):545–7. 10.1188/14.ONF.545-547. 10.1188/14.ONF.545-547 25158659

[31] Gupta J, Betron M, Brown J, Morgan R (2020). Mainstreaming gender into global health programming to improve women's health. Health Care Women Int.

[32] Kågesten A, Chandra-Mouli V (2020). Gender-transformative programmes: implications for research and action. Lancet Glob Health.

[33] Ved R, Scott K, Gupta G, Ummer O, Singh S, Srivastava A (2019). How are gender inequalities facing India’s one million ASHAs being addressed? Policy origins and adaptations for the world’s largest all-female community health worker programme. Hum Resour Health.

[34] Najafizada SAM, Bourgeault IL, Labonte R (2019). A gender analysis of a national community health workers program: a case study of Afghanistan. Glob Public Health.

[35] Okali C (2006). Linking livelihoods and gender analysis for achieving gender transformative change food and agriculture Organization of the United Nations (FAO).

[36] Uzondu CA, Doctor HV, Findley SE, Afenyadu GY, Ager A (2015). Female health workers at the doorstep: a pilot of community-based maternal, newborn, and child health service delivery in northern Nigeria. Glob Health Sci Pract.

[37] Pettifor A, Lippman SA, Gottert A, Suchindran CM, Selin A, Peacock D (2018). Community mobilization to modify harmful gender norms and reduce HIV risk: results from a community cluster randomized trial in South Africa. J Int AIDS Soc.

[38] Mumtaz Z, Salway S (2005). ‘I never go anywhere’: extricating the links between women's mobility and uptake of reproductive health services in Pakistan. Soc Sci Med.

[39] Hussain TM, Smith JF (1999). Women's physical mobility in rural Bangladesh: the role of socio-economic and community factors. Contemporary South Asia.

[40] Dhatt R, Theobald S, Buzuzi S, Ros B, Vong S, Muraya K (2017). The role of women's leadership and gender equity in leadership and health system strengthening. Global Health, epidemiology and. Genomics.

[41] Evans A (2015). Gender sensitisation in the Zambian Copperbelt. Geoforum.

[42] Ghimire-Bastakoti K, Bastakoti RR (2006). Social inclusion in community forestry: why women are frequently excluded from decision-making and leadership in Nepal. Women's Global Connection Conference: Building Community Leadership in Global Society.

[43] UN Women (2017). UN Women Bangladesh: UN Women Asia and the Pacific.

[44] Westeneng J, d’Exelle B (2015). How economic empowerment reduces women’s reproductive health vulnerability in Tanzania. J Dev Stud.

[45] Reed E, West BS, Salazar M, Monroy RV. Economic empowerment to improve sexual and reproductive health among women and girls. Global Perspectives on Women's Sexual and Reproductive Health Across the Lifecourse. Switzerland: Springer; 2018. p. 297–315.

[46] Najafizada SAM, Bourgeault IL, Labonté R. A gender analysis of a national community health workers program: a case study of Afghanistan. Global Public Health. 2018:1–14. 10.1080/17441692.2018.1471515. 10.1080/17441692.2018.1471515 29733267

[47] Kok MC, Kane SS, Tulloch O, Ormel H, Theobald S, Dieleman M (2015). How does context influence performance of community health workers in low- and middle-income countries? Evidence from the literature. Health Res Policy Syst.

[48] Glick P (2008). What Policies will Reduce Gender Schooling Gaps in Developing Countries: Evidence and Interpretation. World Dev.

[49] Kardam N (1995). Conditions of accountability for gender policy: the organizational, Political and Cognitive Contexts. IDS Bulletin.

