# Supplementary figures and images for: Opportunities for gender transformative approaches in a community-based drowning reduction program in Bangladesh
Source: Int J Equity Health. 2020 Jul 1;19:108. doi: 10.1186/s12939-020-01226-z (PMC7329458; doi:10.1186/s12939-020-01226-z)

## Appendix A: Organisational chart of the Anchal program


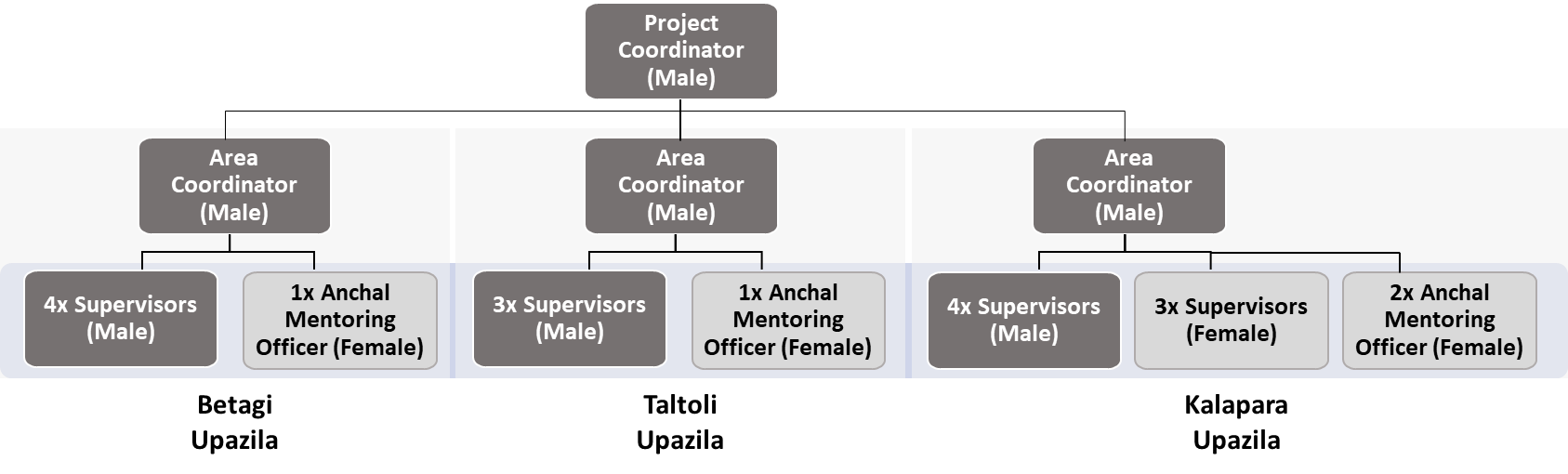

Supplement: Supplementary file 1 — Additional file 1. Organisational chart of the Anchal program. [file 12939_2020_1226_MOESM1_ESM.docx]

## Appendix B: Logic model for the Anchal program


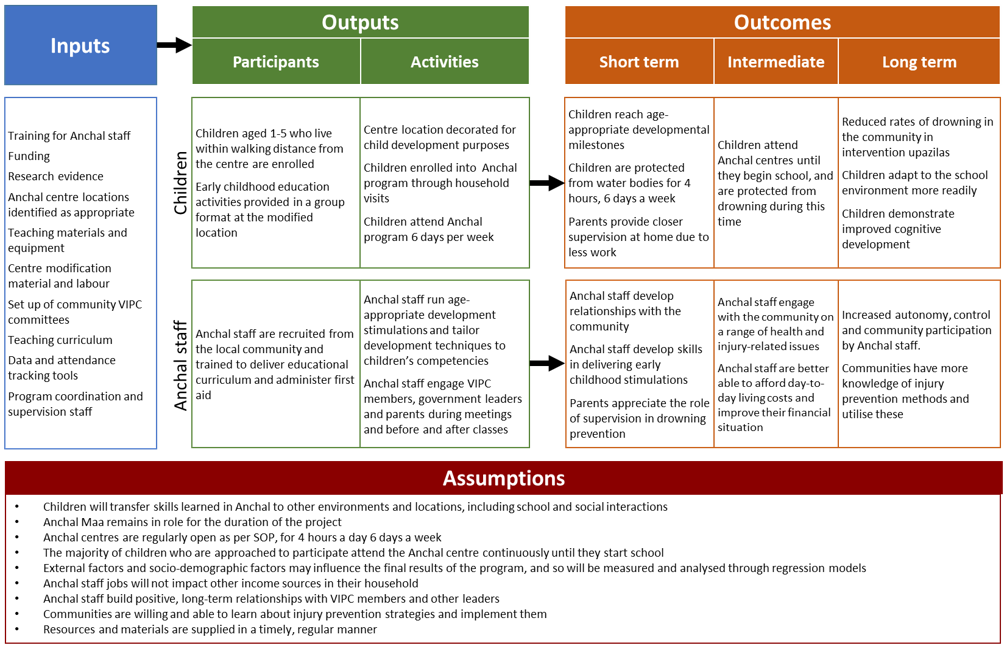

Supplement: Supplementary file 2 — Additional file 2. Logic model for the Anchal program. [file 12939_2020_1226_MOESM2_ESM.docx]
