# Supplementary material for: Opportunities for gender transformative approaches in a community-based drowning reduction program in Bangladesh
Source: Int J Equity Health. 2020 Jul 1;19:108. doi: 10.1186/s12939-020-01226-z (PMC7329458; doi:10.1186/s12939-020-01226-z)
Supplement: Supplementary file 4 — Additional file 4. Codebook [file 12939_2020_1226_MOESM4_ESM.docx]

## Appendix D: Codebook

Nodes – Phase 1

| Name |
| --- |
| 1. Access to Resources |
| Access to information |
| Clothing |
| Financial |
| Free stuff |
| Further education |
| Independence to access resources |
| Mothers free time |
| Phone |
| Schooling for kids |
| Support family business |
| Tuition provision |
| 2. Beliefs, knowledge and attitudes |
| Anchal M and A training |
| Anchal mobility |
| Confidence |
| Drop outs |
| Effect on self and sense of value |
| Family response |
| Gender of AMO |
| Girls v Boys learning |
| Honour and status |
| Husband as earner |
| Identity |
| Love for children |
| Male supervisors |
| Mothers as main child carer |
| Mothers role as caretaker |
| Singing and dancing |
| Supervisor gender |
| Travel by bike |
| Use of education |
| VIPC representation |
| 3. Practices and Participation |
| Communication skills training |
| Community participation |
| Domestic violence |
| Domestic work |
| Father Involvement |
| Female staff with community |
| Female supervisor in community |
| Future employment |
| Home decision making |
| Injury reporting |
| Lack of work experience |
| Other employment |
| Other organisation |
| Physical weakness |
| Providing support |
| Safety |
| Shopping for home |
| Supervision of children |
| Women in VIPC |
| 4. Legal Rights and Status |
| AC |
| AMOs |
| Supervisors |
| 5. Power |
| Ability to work as Maa |
| Identity |
| Oversight over finances |
| Persuasion power |
| Relationship between husband and wife |

Nodes – Phase 2

| Name |
| --- |
| 1. Access to resources |
| Both males and females helpful to women |
| Education is empowering |
| Encourages other women to get educated |
| Finances |
| AA effect is less |
| AA hide salary for honour as so low |
| AA money goes to husband as can't do much with it |
| Can’t buy much with 1000 |
| Family value less |
| Taking time away from other activities |
| Animals |
| Education expenses |
| Faster response when money needed |
| Financial Independence |
| Financial management |
| Home modifications with money |
| Invest in something that makes money |
| More say in finances of home |
| Relief from financial stress |
| Support family financially |
| More relationships with other community women |
| New opportunities and connection |
| Travel for female supervisors |
| Women can’t access many jobs or leave village |
| 2. Knowledge, beliefs and perceptions |
| Acceptable because at home |
| AMs cannot stay at night |
| Change in perception around working women |
| Communication skills |
| Dancing |
| Community engagement to change minds |
| Community watching activities |
| Husbands needing engagement |
| Less change in fathers mindsets |
| Making use of education |
| Negative rumours |
| New identity |
| Perceptions of housewives capabilities |
| Pride and honour |
| Valued at home more |
| Widowed or separated women |
| 3. Legal Rights and Status |
| Familiarity |
| Safety in working at a school v domestic maid |
| Learn better language |
| Mothers working so can't teach children |
| School readiness |
| Toys |
| Kids don't enjoy without toys |
| 5. Power |
| Change of power after marriage to husband |
| More power if worked before already |
| Need in-law's approval |
| Subverting others' power |
| 4. Practices and participation |
| Appeasement of family with gifts |
| Attendance at parent meetings |
| Domestic work |
| Management of duties |
| Particular benefit for mothers as do household work |
| Primary duty is to the home |
| Sharing of duties with husband |
| Sharing of work |
| Training difficulty |
| View that the work isn't much |
| Easier accommodation for female DCs |
| Family support necessary |
| Gender of supervisor or AMO |
| AM discomfort and engagement |
| Cover for each other |
| Engagement with leaders |
| Engagement with parents and community |
| Female supervisor for conservative areas |
| Night time mobility and security |
| Transport difficulties |
| Washroom problem |
| Highly conservative communities |
| Men question female staff |
| Parda system preventing parents dropping off |
| Women don't attend parent meetings |
| Home decision making |
| Marriage leads to drop out |
| Mobility |
| Increased mobility |
| No problems moving around |
| Reduced community criticism |
| Travel anxiety |
| Relationships with community |
| Use of education |
| VIPC |
| Females needed to interact with girls |
| Low females |
| Male v female contribution |
| Males give less time |
| More females support each other |
| Wearing veil in training |
| Women's role as primary child carer |
